# A Conceptual Framework for Understanding Variability in Student Perceptions

**DOI:** 10.3389/fpsyg.2021.725407

**Published:** 2021-10-20

**Authors:** Bilge Gencoglu, Michelle Helms-Lorenz, Ridwan Maulana, Ellen P. W. A. Jansen

**Affiliations:** Department of Teacher Education, University of Groningen, Groningen, Netherlands

**Keywords:** student perception, teaching quality, value orientation, value congruence, variety of perception, social desirability

## Abstract

Student perceptions using surveys are frequently used to measure student perceptions of teachers’ teaching quality in secondary and higher education. Research shows that the variance in student perceptions exists within a class and between countries. However, the influence of individual- and cultural-level factors on the variance of student perceptions is less well studied. More insights are needed to understand the mechanisms underlying the variance in student perceptions in-depth. Insights into determinants of student perceptions of teaching quality could become valuable toward understanding school-related outcomes. A conceptual framework is put forward in this study to enhance our understanding of manifestations of student perceptions of teaching quality. It is suggested that value orientations at the individual- and cultural-level as well as social desirability may play a role in understanding student perceptions of teaching quality. Understanding students’ individual and collective perceptions of teaching quality can contribute to teachers’ sense-making of their student evaluations. It is argued that this understanding could contribute to enhancing the development of teaching quality and ultimately education quality.

## Introduction

Student perceptions of teachers’ teaching quality are important for students’ school-related outcomes, such as academic motivation and engagement (Maulana et al., 2016; Maulana and Helms-Lorenz, 2016; Inda-Caro et al., 2019). Although the importance of student perceptions for school outcomes is largely acknowledged by scholars, more insights are needed to understand the factors that influence student perception besides the teacher’s behavior in the classroom. Student perceptions of the same teacher’s teaching quality vary within a class (between-student variations; Maulana et al., 2015). The variety in student perceptions of the same teacher suggests that there are individual factors affecting student perceptions. Similarly, student perceptions vary across countries (André et al., 2020). The variety in student perceptions within and across countries may be an indication that there are cultural factors affecting student perceptions. This conceptual paper explores value orientations as an underlying individual- and cultural-level factor explaining variations in student perceptions.

Explorations of the variations in student perception have the potential to enrich our understanding of the nature of the information contained in student ratings. However, variance among students within the same classroom is frequently treated as an error or nuisance variation (e.g., Meade and Eby, 2007). In other words, it is assumed that “each student would assign the same rating, such that the responses of students in the same class would be interchangeable” (Lüdtke et al., 2006, p.217). Compared to the immense body of research focusing on the mean levels of aggregated student ratings at the class-level, much less attention has been paid to the degree to which students within a class agree in their perceptions (within-class variety, e.g., Lüdtke et al., 2006; Schweig, 2016). These studies investigate whether within-class variability^1^ has predictive power for learning and achievement outcomes (Schweig, 2016; Schenke et al., 2017, 2018; Bardach et al., 2019b, 2021).

The variety of perceptions can be delineated by the psychological processes described by scholars of person perceptions. The rational-emotive behavior (REB) model (Ellis, 1962) describes how the interpretation of the same observed behavior by two different people can lead to different perceptions. One of the primary tenets of the REB model is that thoughts, feelings, and behaviors interact and affect each other (Ellis, 1962). Ellis (1991) asserts that thinking affects and creates feelings and behaviors; at the same time, emotions influence thoughts and behaviors, and behaviors have an impact on thoughts and feelings. Consequently, if one of these processes is altered, the other processes are influenced dynamically as well (Banks and Zionts, 2009). Because individual experiences and reactions to experiences are different, the perceptions will differ, too. The hypothesis of equal perceptions of teaching quality is highly unlikely given the psychological mechanisms put forward by Ellis’s model.

Ellis (1974) posits an ABC theory of disturbance. A is defined as an Activating event or activating experience; B is defined as a Belief system; and C is defined as emotional and behavioral Consequences. These consequences may either be (1) healthy as a result of rational perceptions or (2) self-defeating as a result of irrational or distorted perceptions (Banks and Zionts, 2009). Based on REB, Clawson (2012) proposes that observed behavior is compared to a personal set of values, assumptions, beliefs, and expectations about the way the world is or should be. This comparison leads one to make conclusions and interpretations about the observed behavior, which determines the perception of what is observed. The role of beliefs is hypothesized as mediating the relationship between external events and emotional consequences. Irrational beliefs lead to disturbing emotions, such as depression, fear, anger, and negative behavioral reactions, such as withdrawal and impulsivity (Bernard, 1990). Rational beliefs, on the other hand, generally result in moderate emotions that foster goal attainment and life satisfaction (Smith, 1982). In other words, an individual’s emotional and behavioral response is thought of as caused by an external event, yet it is the result of a combination of an external event and the processing of the information by the individual’s belief system (Ellis, 1962).

Applying the REB model to student evaluations of teaching quality implies that evaluations do not only consider the observed external event, that is, teaching quality, but also incorporate the processing of the information by students, that is, personal set of belief systems. A value-belief-norm theory suggests that individual values lead to beliefs, which in turn help to form personal norms (Stern et al., 1999; Stern, 2000). Given that values operate to shape beliefs and norms, they are also involved in student evaluations of teaching quality. In this conceptual paper, values are explored to understand student’s individual and collective perceptions of teaching quality. Student evaluation of teaching quality is treated as an instance of perceptions and value orientations as underlying reasoning behind perceptions.

Teaching quality can be conceptualized in many different ways (Helms-Lorenz and Visscher, 2021). Scheerens (2016) postulated a distinction between the pro-active (the preparation activities and prerequisites involved before a lesson is executed), interactive [the (in)visible interactions during the lesson], and retro-active (the evaluation of the conducted lesson and of student learning after the execution of the lesson) aspects of teaching. Although manifest teaching behavior in the classroom is viewed as a proxy of teaching quality reflecting the three aspects of teaching (Helms-Lorenz and Visscher, 2021), student perceptions of teaching quality address the classroom teaching behavior during the lesson because student perceptions are linked with teachers’ act in the classroom. Various frameworks have been developed to study classroom teaching behavior and its relation to students’ school-related outcomes (Creemers, 1994; Scheerens et al., 2007; van de Grift, 2007; Creemers and Kyriakides, 2008; Pianta et al., 2008; Hattie, 2009; Pianta and Hamre, 2009; Sammons and Bakkum, 2011; Danielson, 2013). Although these frameworks lead to several measurement instruments varying in terms of structure and their main underlying models and conceptualizations, at least six teaching behavior domains were revealed showing a relationship with students’ learning and outcomes: Providing a Safe and Stimulating Learning Climate, Efficient Classroom Management, Clarity of Instruction, Activating Learning, Adaptive Teaching, and Teaching Student Learning Strategies. Also, teacher-student interpersonal relationship has been shown to be an important determinant of the learning processes of students (van Tartwijk et al., 1998; den Brok, 2001). For example, the framework developed by Hamre et al. (2013), which focuses on teacher-student interactions, proposes a three-domain structure, namely, emotional supports, classroom organization, and instructional supports. Although each framework has significant contributions in the literature, choosing a specific framework of teaching quality might narrow down the scope of our proposed framework. It is thus noteworthy to mention that the conceptualization of teaching quality is not restricted to a specific theoretical framework.

In the following sections, values are defined and individual value orientations are reviewed. The concept of value (in)congruence is presented with regard to interactions resulting from similar and dissimilar values and applied to student perceptions of teaching quality. Then, cultural value orientations are put forward and applied to student perceptions of teaching quality. It is followed by unpacking the concept of social desirability, which addresses the tendency to share similar ideas with others. By proposing a conceptual framework, we conclude that value orientations, value congruence, and social desirability provide rich background information that might allow predictions and understanding of the student perceptions variations within and between cultures.

## Values

Values are defined as deep, enduring, and guiding principles that influence people’s behavior (Rokeach, 1973; Schwartz, 1992; Parashar et al., 2004). They are desirable, abstract goals that guide beliefs, attitudes, perceptions, and behaviors (Rokeach, 1973; Schwartz, 1992) that reflect the way one wants the world to be. While norms, attitudes, and specific goals depend on specific situations, actions, or objects, values transcend specific situations (Schwartz, 1992). Unlike traits, orientations, and interests, values serve as criteria or standards to provide social justification for choices and behaviors (Roccas et al., 2002; Sagiv, 2002). Values help to shape personal and collective preferences about what is important. In other words, values guide individuals and societies about how to act in particular situations.

Schwartz (1992, 1994) developed two different value theories, one regarding individual differences in values and the other regarding cultural differences. The first theory assumes that values are ordered by subjective importance and form a unique system of value orientations at the individual level (Rokeach, 1973; Schwartz, 1992). The second theory assumes that values are ordered by hierarchical social systems, reflecting the value orientations at the cultural-level (Schwartz, 2006). The two proposed orientations reflect that the culture-level processes are different from individual-level processes (Schwartz, 1999; Fischer et al., 2010). By doing so, Schwartz followed the same tradition established by Hofstede (1980) who argued that individual and cultural levels are not isomorphic (Fischer et al., 2010). Schwartz proposed different structures at the two levels arguing that the characteristics that discriminate among societies and those that discriminate among individuals are unlikely to be the same (Schwartz, 1994). In the following sections, we explore the possible influence of individual and cultural values on student perceptions.

### Individual Values

**Figure 1:**
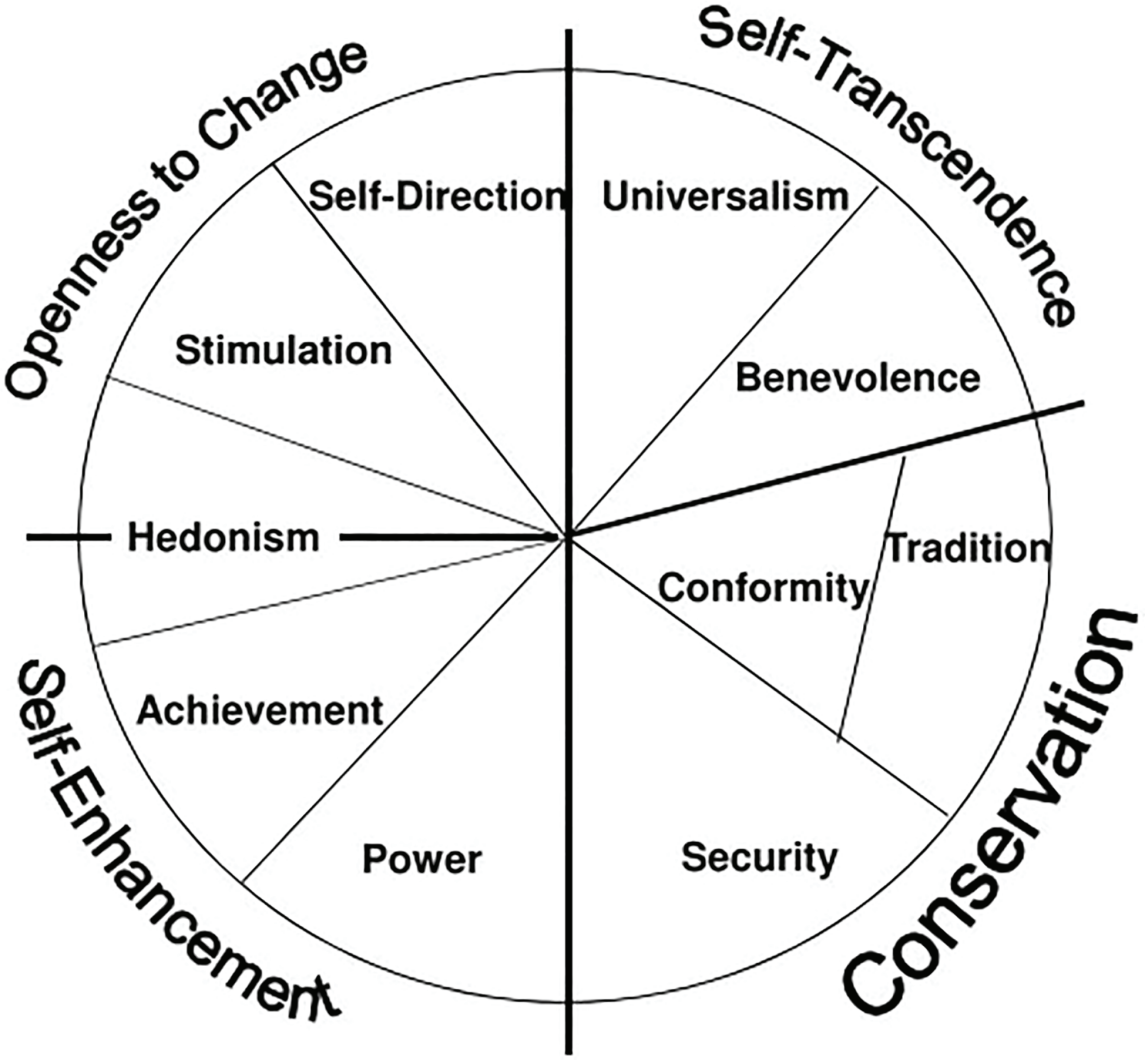
The Circular Structure of Values Relations and Inter-Relatedness. This figure represents the theoretical model of relations among ten types of value. From Schwartz (2012b). The figure has been used with the permission of the author.

At the individual level, Schwartz’s (1992) postulates ten basic universal values. The circular structure of values put forward by Schwartz depicts the relations between values (see Figure 1). In the circle, congruent values are adjacent to one another, whereas conflicting values are in opposing directions from the center. The values have bipolar dimensions: (1) self-enhancement versus self-transcendence and (2) openness to change versus conservation. Self-enhancement values (power, achievement) emphasize and legitimize the pursuit of one’s own interests, whereas self-transcendence values (universalism, benevolence) legitimize the pursue of the welfare of others. Openness to change values (self-direction, stimulation) encourage change, new ideas, and experiences, whereas conservation values (security, tradition, conformity) emphasize maintaining the establishment of shared rules and avoiding threat. Hedonism values share elements of openness and self-enhancement. Values of self-transcendence and conservation are externally focused and represent the association with others. In contrast, values of self-enhancement and openness to change are self-focused and represent the expression of personal interests and characteristics (Schwartz, 2012b). In fact, these ten values have been split into finer subparts which construct 19 values (Schwartz, 2017). However, these subparts are not included in this paper for the sake of simplicity and because these ten values already construct a broad and comprehensive understanding of values.

The value theory of Schwartz suggests that intra-individual value importance change occurs in a coherent manner (Schwartz, 1992). This organized pattern argues that if one value increases in importance while the opposing value remains stable, it may cause uneasy feelings, inner dissonance due to the inconsistency (Daniel and Benish-Weisman, 2019). Therefore, it is expected that adjacent values change in the same direction and values on the opposing side of the circle simultaneously change in the opposite direction (Bardi et al., 2009). Bardi et al. (2009) examined intra-individual change in value structures among adolescents longitudinally. They found that increases in the importance of each value were accompanied by those of compatible values and decreases in the importance of opposing values. Specifically, adolescents who wish to stand on their own, which corresponds to the value of openness to change, feel inner-conflict because of their simultaneous desire to conform to their peer group, which corresponds to the value of conservation. In a similar manner, adolescents who strive for dominance in their group, which corresponds to the value of self-enhancement, may feel less inclined to tolerate variety among peers, which corresponds to self-transcendence. Such distress and conflict lead to changes in values hierarchies. In an experimental study, it was shown that increases in the manipulated values were accompanied by decreases in opposing values that were not manipulated (Maio et al., 2009).

Apart from inner value conflict, contradicting values occur interpersonally, too. For example, students and teachers might have opposing values, which leads to emotional and behavioral consequences among them. The emotional and behavioral consequences are the result of student and teacher perceptions. In other words, interpersonal value synergy or a “click” between teachers and students potentially depends on value congruence. In order to understand how a teacher and a student can be a good match, it is necessary to consider the value congruence.

### Value Congruence

Individuals behave congruently with social events and social environments that are in line with their personal values. Lack of alignments or conflict leads to adverse attitudinal and behavioral expressions between individual and/or collective value orientations (Vauclair and Fischer, 2011). Person-environment fit (P-E fit) in the job setting can be “broadly defined as the compatibility between an individual and a work environment that occurs when their characteristics are well matched” (Kristof-brown et al., 2005, p. 281). In a broader sense, it can be defined as the match, congruence, similarity, or correspondence between the person and the environment. The mechanisms of person-environment fit function through the process of need fulfillment (Rice et al., 1989; Van Vianen, 2000). Achieving person-environment fit is a way to have individual needs met. The need fulfillment processes which are related to experiencing person-environment fit suggest that if needs are satisfied, individuals will experience more positive attitudes. On this basis, theories, such as the social comparison theory (Festinger, 1954), the balance theory (Heider, 1958), the similarity-attraction paradigm (Byrne, 1971), and the attraction-selection-attrition framework (Schneider, 1987), suggest that people have a fundamental need for consensual validation of their perspectives, which can be met by interacting with similar others. For example, individuals are attracted to careers that match with their values, therefore in the situation of a mismatch dropout rates increase (Holland, 1985, 1997; Schneider et al., 1995; Knafo and Sagiv, 2004). Consequently, people in a congruent environment will develop or benefit more than those in an incongruent environment (Feldman et al., 2001). When P-E fit is applied to the classroom and educational environment, students might have more benefits in a classroom that provides a congruent environment and from a teacher who performs the expected roles that fit the environment, that is, who provides a good quality teaching that helps students learn.

Researchers not only investigate the congruency of the environment but also the extent to which one’s values are congruent with those that are salient in a particular context and among significant others, such as with a job manager or parents. Along these lines, the similarity-attraction paradigm shows that individuals are attracted by others who share similar characteristics (Byrne, 1971). Similarity binds people together because similar values facilitate communication and reduce uncertainty in interpersonal relationships (Kalliath et al., 1999; Edwards and Cable, 2009). Numerous studies have examined student-teacher congruence, particularly matches in student-teacher gender, race (Oates, 2003; Bates and Glick, 2013), thinking style (Zhang, 2006), beliefs (Polat, 2009), and expectations (Turner, 2009), and its relationship with learning achievement and test performance (e.g., Oates, 2003). The congruence in underlying characteristics (e.g., beliefs, expectations, values) might function differently from congruence in appearance (e.g., gender, race) because the congruence in appearance is more explicit and visible than the congruence in underlying innate characteristics. Westerman et al. (2002) illustrated that value congruence is a predictor of student satisfaction. Value congruence creates an environment in which individuals can freely express personal values and attain shared goals supported in the environment (Sagiv and Schwartz, 2000). This value match influences students’ academic achievement because values shape lives, influence actions, gives expression to underlying beliefs (Rokeach, 1973); thus, it reflects to what extent students feel close to their teachers and how much students like their teachers. Given the value congruence, situations allowing for opposing student-teacher values may lead to more variations in student perceptions compared to situations that nurture value congruence.

### Cultural Values

Learning culture represents a set of shared beliefs, values, and attitudes favorable to learning. Students’ learning is influenced by learning culture at the school level. Schools’ learning culture is influenced by higher cultural contexts at the regional and national levels. In this respect, cultures operate to shape the environment, behavior, and minds of their members. Value orientations at the cultural level provide a higher-order frame for understanding differences in student perceptions across cultural settings. In this paper, Schwartz’s (1994, 1999, 2006) cultural-level value orientations are consulted because it is acknowledged that cultural-level processes are distinct from individual-level processes. These cultural-level value orientations are considered as “the cultures of the national groups” (Schwartz, 1999, p. 25).

**Figure 2:**
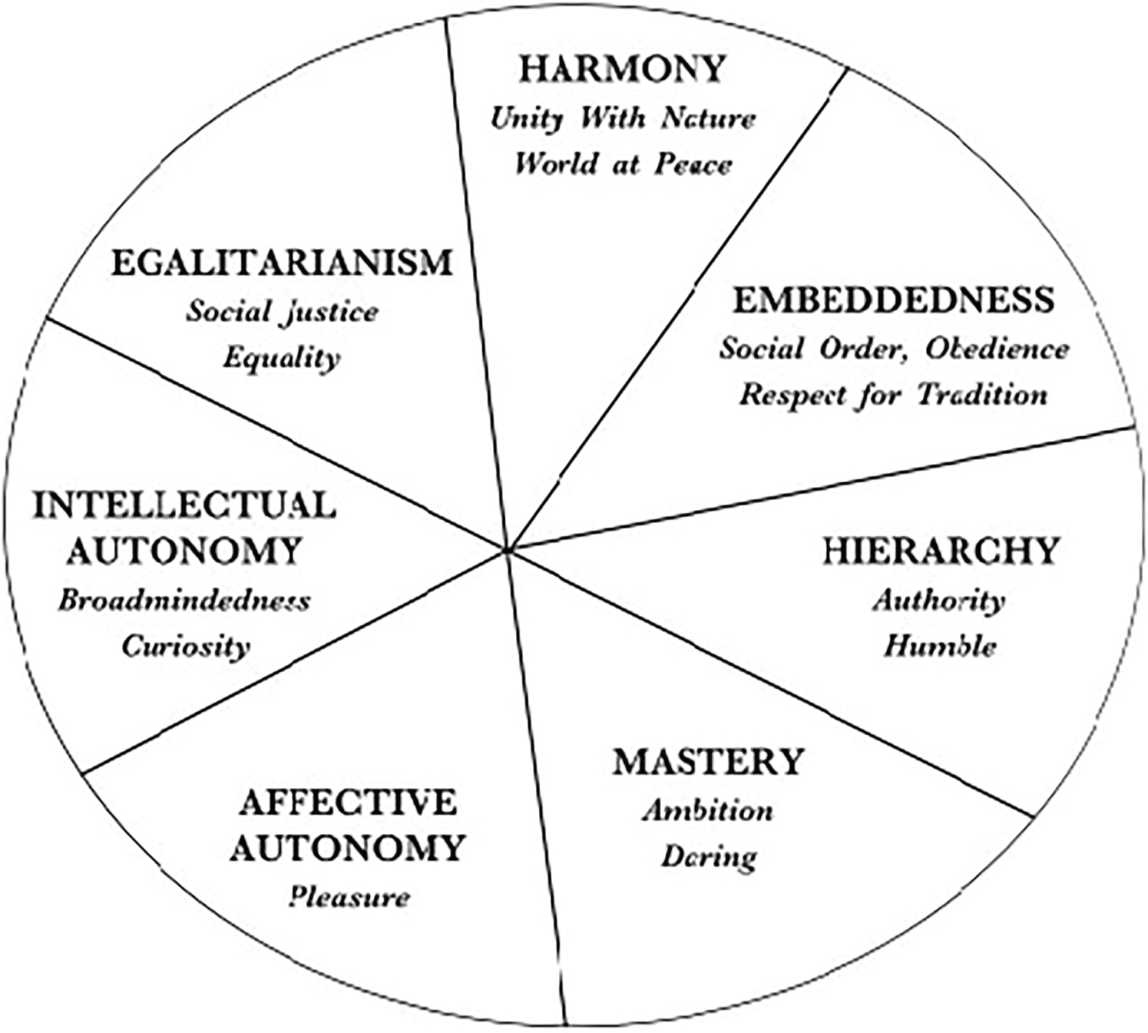
Structure of Culture-Level Value Types. This figure represents the theoretical model of relations among ten types of value at the cultural level. From Schwartz (2006). The figure has been used with the permission of the author.

At the culture level, seven cultural value orientations can be captured along three distinct dimensions (Schwartz, 1994, 1999, 2006). Schwartz argues that there are three basic issues in every society: (1) to what extent an individual belongs to a group, (2) how to preserve the social fabric, and (3) how to relate to the social world. As seen in Figure 2, bipolar value orientations are formed, and cultural influences on which pole of the orientation are emphasized in society. Regarding the first societal issue, autonomy vs. embeddedness, autonomy partitions into affective autonomy and intellectual autonomy, which refers to values that encourage people to pursue positive experiences for themselves as well as their own ideas and intellectual aspirations. In autonomous cultures, individuals are encouraged to think, feel, and act as unique persons. The opposite pole, embeddedness, encourages traditional order, shared goals, ways of living, and the maintenance of the status quo is seen as a priority. The second societal issue produces the value types of hierarchy vs. egalitarianism. The hierarchy represents the hierarchical social order and unequal resource allocation; in contrast, egalitarianism emphasizes equality, mutual concern, and cooperation for everyone’s welfare. The last societal problem produces the values of harmony vs. mastery. The former reflect societal discourses in which the social and natural world is acknowledged as it is, and fitting in harmoniously is emphasized. Individuals strive for a world at peace and the protection of the environment. The latter depicts the active control of the social and natural environment through self-assertion values. Individuals are ambitious, seek success, and competence in order to attain group or personal goals.

Similar to the individual level of value orientations, poles of the cultural value orientation tend to conflict with each other. Prevailing cultural value orientations are assumed as ideals that promote coherence, whereas incompatible values are likely to generate tension, elicit criticism, and pressure to change (Schwartz, 2006). Examples thereof are examined in the frame of prejudice and aggression toward an outgroup with different value orientations (Struch and Schwartz, 1989). In a society where hierarchical relationships are legitimate, individuals tend to mark off in and outgroups. In contrast, in cultures higher on egalitarianism, emphasizing the equality of all people in the world, members of outgroups are less likely to be devalued (Tajfel and Turner, 1979; Schiefer et al., 2010).

**Figure 3:**
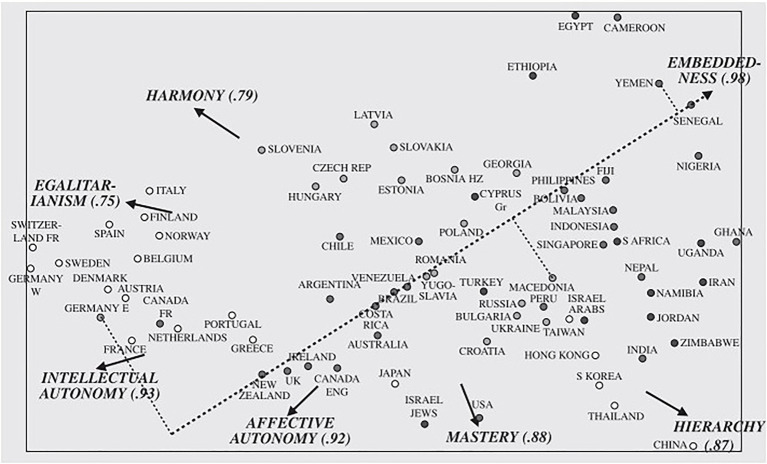
Cultural Value Orientations from 76 National Groups. The figure represents cultural value orientations from 76 national groups based on the combined teacher and college student samples. From Schwartz (2006). The figure has been used with the permission of the author.

Applied to the educational setting (see Table 1), it is hypothesized that in certain cultures where, for example, harmony is emphasized it is more probable that students and teachers will share the same harmonious values to a greater extent compared to the opposite pole. This will lead to less openness to communicate about differences. Therefore, in these communities where more alignment occurs on harmony, we expect less variety in student perceptions with regard to teaching quality. Similarly, in certain cultures where, for example, embeddedness is emphasized, less variety in student perceptions with regard to teaching quality is expected because in these cultures individuals are viewed as entities embedded in the collectivity with shared goals. Along the same line, if hierarchy is emphasized in a culture, it is more probable that students will consider teachers at a higher-order level and feel responsible to obey. Therefore, it is more probable that they will share similar opinions about teachers and extreme opinions will be less encouraged compared to the opposite pole. This will again lead to less variety in student perceptions with regard to teaching quality. Based on the studies in which the groupings of nations were depicted (see Figure 3; Schwartz, 1999, 2006), less student perceptions variety in, for example, Slovenia, Singapore, Indonesia, and South Korea, is expected because these cultures have adopted the pole of harmony, embeddedness, and hierarchy.

**Table 1:** The relationship between cultural values, student perception, and social desirability.

Cultural values	Manifestations of student perception	Social desirability
Harmony	More congruence, less variability of perception	More impression management, less self-deceptive positivity
Embeddedness		
Hierarchy		
Mastery	Less congruence, more variability of perception	Less impression management, more self-deceptive positivity
Affective autonomy		
Intellectual autonomy		
Egalitarianism		

In contrast, in certain cultures where, for example, mastery is prioritized, self-assertion is seen as individual ambition and competence. Therefore, because individual opinion differences are expected to be openly discussable, more variety in student perceptions is to be expected. Similarly, if autonomy is prioritized in a certain culture, an individual’s unique thinking, feeling, and acting are encouraged, which is expected to lead to a variety of student perceptions. Along a similar line, in cultures where, for example, egalitarianism is prioritized, embracing different opinions and value orientations is more probable. In these environments, students and teachers are hypothesized not to hesitate to express own value orientations, leading to tolerance of diverse values orientations, and more variety in student perceptions. Based on groupings of nations (see Figure 3; Schwartz, 1999, 2006), more student perceptions variety in, for example, the United States, Canada, The Netherlands, and Spain, is expected because these cultures have adopted the pole of mastery, autonomy, and egalitarianism values.

One of the important features of cultural value orientations is that cultural values shape and justify individual and group beliefs, actions, and goals (Schwartz, 2006). In every layer of society, from child-rearing and everyday practices to institutional regulations and policies, there are traces of cultural values emphasized in the society. Indeed, it is inevitable to expect the projections of cultural value orientations in the classroom and educational settings. For example, schools in primary and secondary education in The Netherlands need to pay attention to citizenship education. This education ensures that students develop social and societal competencies and respect for differences in every aspect such as religions, beliefs, political views, and sexual orientations. Regardless of these differences, schools need to ensure an environment in which students and staff feel safe and accepted. Students learn to listen to each other, determine their opinion and express it constructively, as well as, notice and respect different opinions. These education practices coincide with the emphasis of the cultural orientation, which is hypothesized to be at the pole of mastery, autonomy, and egalitarianism value for The Netherlands (Schwartz, 2006, 2014). It is therefore important to recognize the nested nature of education.

Another important feature is that cultural value orientations can change, yet the change is slow (Schwartz et al., 2000). Although cultural orientations can persist over hundreds of years, they change gradually. The augmentation of wealth and contact with other cultures by globalization, the massive effect of the pandemic, and the advancement in technology, all together may lead to change in cultural value orientations. Due to this slow and gradual change, a change in educational practices and policies is also to be expected.

## Social Desirability

Understanding how socially desirable responding (SDR) impacts student self-reporting practices might be useful to understand the influence of shared values on student perceptions (Schwartz et al., 1997). SDR (Crowne and Marlowe, 1960) refers to the tendency to respond in a manner that strengthens social approval instead of reflecting one’s true feelings. Concerns of social desirability may be inevitable in the case of values because values are the goals and preferences individuals consider socially desirable. Respondents are likely to respond in a biased manner to the degree that certain values are strongly encouraged within the social environment in certain social contexts (Fisher and Katz, 2000). In contrast, values that are of marginal importance are less likely to influence responses, meaning that the motivation of social desirability bias will be weak.

Paulhus (1991) divides two factors of social-desirability bias. The first factor, *impression management*, is the desire to represent oneself in a socially conventional way. Individuals who have higher scores on impression management tend to be more sensitive to social influence. It was found that impression management is more related to values highlighting the importance of social harmony (i.e., conservation and self-transcendence) rather than to those characterized by a personal focus (i.e., openness to change and self-enhancement; Danioni and Barni, 2020). The second factor, *self-deceptive positivity*, reflects a favorable self-presentation. Individuals who score higher on self-deceptive positivity hold values characterized by a personal focus. A positive link between self-deceptive positivity and self-enhancement values and a lack of relationship between self-deceptive positivity with conservation and self-transcendence demonstrates that self-deceptive positivity is characterized by a focus on the self (Danioni and Barni, 2020).

For an individual to indicate SDR, the individual needs to have some knowledge of what would be desirable in the corresponding cultural context (i.e., social-norm intelligence; Bou Malham and Saucier, 2016). Hence, SDR involves implicit reference to culturally shared norms, standards, and values. Although evidence is mixed as to whether individuals in different cultures exhibit SDR to the same extent, much research supports cross-cultural differences. For example, it was argued that SDR can be an adaptive response strategy in certain situations, in which strong norms are shared around a particular issue and are learned through socialization (Ross and Mirowsky, 1984). Research shows that individuals in collectivistic cultures tend to have higher impression management and lower self-deceptive scores, whereas individuals in individualistic cultures tend to have higher self-deceptive scores (Lalwani et al., 2006). For example, it was shown that Hong Kong participants scored higher on impression management than did US participants, whereas US participants scored higher on self-deceptive than did Hong Kong participants (Lalwani et al., 2009). Such findings suggest that impression management and self-deceptive positivity reflect different cultural settings. Given the relationship between SDR and cultural contexts, SDR can be interpreted as cultural consonance to maintain person-environment or person-group congruence (Bou Malham and Saucier, 2016).

In the school context, peers play a decisive and critical role in students’ behaviors and attitudes. Through friendship, peers influence student’s academic functioning (Newcomb and Bagwell, 1995; Rambaran et al., 2017). This academic functioning includes involvement in school (Kindermann, 2007), motivation (Wentzel et al., 2010; Molloy et al., 2011), and reading achievements (Cooc and Kim, 2016). As seen in Table 1, it would be expected that the variety of student perceptions changes due to the influence of peers concerning SDR depending on cultural contexts. Specifically, in certain cultures where the pole of harmony, embeddedness, and hierarchy is emphasized, it is hypothesized that students are more likely to engage in impression management and less likely in self-deceptive positivity to maintain good relationships with others compared to the opposite pole. In these cultural settings, high impression management would likely serve the function of group harmony, which leads to more congruence and less student perceptions variability. In contrast, in certain cultures where the pole of mastery, autonomy, and egalitarianism is emphasized, it is less likely that students engage in impression management and more likely to engage in self-deceptive positivity. In these cultural settings, because self-deceptive positivity is characterized by a focus on the self, individuals are likely to reveal their self-identity by having and expressing their unique and distinctive opinions, which leads to less congruence and more variability among student perceptions.

## Discussion and Concluding Remarks

This conceptual paper explores the theoretical underpinning of value orientations as antecedents of the variation of student perceptions within and across countries and aims to shed light on how value orientations and social desirability may explain variability in student perceptions. In other words, it aims to provide insights into understanding value orientations at the individual and collective level to better understand manifestations of student perceptions. The added value of Schwartz’s value theory promotes understanding of student perceptions through value orientations. Student evaluation of teaching quality is treated as an instance of perceptions, and value orientations as underlying reasoning behind perceptions.

**Figure 4:**
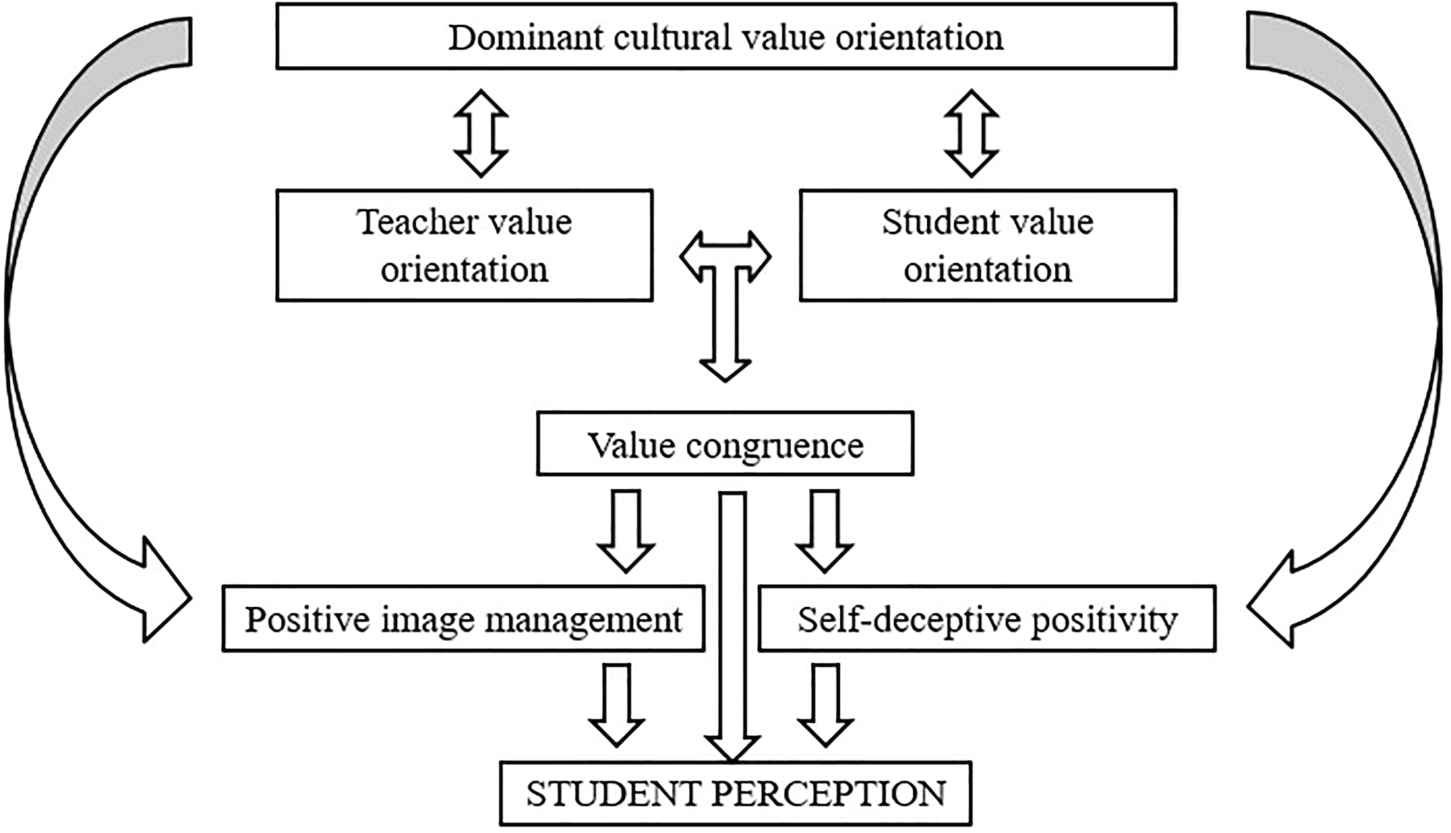
The Proposed Conceptual Framework.

As seen in the proposed conceptual model (see Figure 4), it is postulated that value orientations, value congruence at the individual- and cultural-level, and social desirability may play a role in student perceptions of teaching quality. According to the model, (cultural) value orientations may play a role in influencing every layer of society, including social interactions manifested as value congruence and social desirability. In this sense, teacher’s and student’s individual value orientations could be in interaction with cultural value orientations, which in turn generate value (dis)congruence. Similarly, cultural value orientations could be related to two factors of social desirability, namely, impression management and self-deceptive positivity. Taken together, manifestations of student perception might be influenced by the specific cultural value orientation, value congruence between teacher and student, and social desirability (see Table 1). This model can serve as the theoretical rationale guiding future empirical research for investigating the antecedents of student perception variability of teaching quality. Also, the model can be used to understand the universal reasoning behind the empirical research that investigates the consequences of student perception variability (e.g., Schweig, 2016; Schenke et al., 2017; Bardach et al., 2019a). Empirical research reported that lower levels of student variability are associated with better learning outcomes. For example, it was found that classrooms with higher levels of consensus (i.e., lower levels of variability) regarding the teacher’s ability to effectively control the classroom experienced more effective instruction and vice versa (Schweig, 2016). The model can serve to support teachers toward higher levels of value congruence to enhance a safe and stimulating classroom climate that is fundamental for learning.

This conceptual framework applied to educational settings should be investigated empirically in future research. Besides, individual factors (e.g., age and gender), class context (e.g., learning environment), and school context (e.g., private vs. public) need to be taken into consideration even though they are not portrayed in the conceptual framework. In order to explain the significance of these factors, student age in connection with the development of values can be given as an illustration. With a cross-sectional study, it was revealed that adolescents attribute lower importance to other-focused values and higher importance to self-focused values compared to adults, and values that emphasize autonomy and self-direction peak in late-adolescence (Schwartz, 2012a). Longitudinal studies depict similar findings, regarding students’ values change across the school year (Hofmann-Towfigh, 2007). It was found that adolescents’ self-focused values of self-direction, power, and achievement increase while the other-focused values of self-transcendence, benevolence, and universalism decrease over time. This trend is connected to the natural phenomenon that adolescence is a period of exploration, independence, and identity development. In connection with these developmental processes, it would be expected that young students will be more likely to be influenced by others, that is, peers, because they do not form a self-construal yet. Consequently, it is expected that those students are less likely to have unique opinions on teaching quality, which leads to less variety of student perceptions. In contrast, older students who are at the later stages of their identity and self-construal development are more likely to engage in agentic behavior, which leads to more variety of student perceptions with regard to teaching quality.

From a practical point of view, this paper emphasizes the importance of teachers being aware of and acknowledging different value orientations in the classroom. Students have a personal value orientation, which makes each student unique and special. If teachers assume that students hold the same values with each other and with the teacher, it becomes difficult to create a safe learning environment that supports trust, collaboration, and transparent sharing. At this point, teachers need to be supported to acknowledge individual and cultural diversities. Indeed, teacher education training could incorporate these aspects into their programs, and professional development initiatives could be developed to assist the development of experienced teachers in this respect.

For the sake of educational research and policy-makers, it is also important to emphasize that the extent to which students vary in their perceptions of teaching can be considered a rich source of information when considered from a bigger picture that incorporates cultural orientations. Less variability of student perceptions does not necessarily mean that student perceptions are less valuable, less informative, or less reliable. It simply means that teachers, educational researchers, and policy-makers need to be aware of the influence of shared values, impression management, and self-deceptive positivity reflected in student perceptions of teaching quality. Whether the variability of student perceptions is low or high, students have conceptions about how teaching quality may help or hinder their learning. Therefore, it is essential to emphasize the significance of student perceptions *per se*.

## Author Contributions

Each author has made a substantial contribution to the conception of the work, substantively revised it, and approved the submitted version. BG, MH-L, RM, and EJ: conceptualization. BG: writing – original draft preparation. MH-L, RM, and EJ: writing – review and editing and supervision. All authors contributed to the article and approved the submitted version.

## Funding

This study was part of the Ph.D. project of the first author.

## Conflict of Interest

The authors declare that the research was conducted in the absence of any commercial or financial relationships that could be construed as a potential conflict of interest.

## Publisher’s Note

All claims expressed in this article are solely those of the authors and do not necessarily represent those of their affiliated organizations, or those of the publisher, the editors and the reviewers. Any product that may be evaluated in this article, or claim that may be made by its manufacturer, is not guaranteed or endorsed by the publisher.

## Footnotes

^1^The terms “within-class consensus” and “heterogeneity in student perceptions” were used to specify the degree of within-class variability.
